# Blood flow response to orthostatic challenge identifies signatures of the failure of static cerebral autoregulation in patients with cerebrovascular disease

**DOI:** 10.1186/s12883-021-02179-8

**Published:** 2021-04-09

**Authors:** Clara Gregori-Pla, Rickson C. Mesquita, Christopher G. Favilla, David R. Busch, Igor Blanco, Peyman Zirak, Lisa Kobayashi Frisk, Stella Avtzi, Federica Maruccia, Giacomo Giacalone, Gianluca Cotta, Pol Camps-Renom, Michael T. Mullen, Joan Martí-Fàbregas, Luís Prats-Sánchez, Alejandro Martínez-Domeño, Scott E. Kasner, Joel H. Greenberg, Chao Zhou, Brian L. Edlow, Mary E. Putt, John A. Detre, Arjun G. Yodh, Turgut Durduran, Raquel Delgado-Mederos

**Affiliations:** 1 ICFO-Institut de Ciències Fotòniques, The Barcelona Institute of Science and Technology, 08860 Castelldefels, Barcelona Spain; 2 Institute of Physics, University of Campinas, Campinas, Brazil; 3 Department of Neurology, University of Pennsylvania, Philadelphia, USA; 4 Departments of Anesthesiology and Pain Management and Neurology, University of Texas Southwestern Medical Center, Dallas, USA; 5 Neurotraumatology and Neurosurgery Research Unit (UNINN), Vall d’Hebron University Research Institute (VHIR), Universitat Autònoma de Barcelona, Barcelona, Spain; 6 San Raffaele Scientific Institute, Milan, Italy; 7 Department of Neurology (Stroke Unit). Hospital de la Santa Creu i Sant Pau, Biomedical Research Institute Sant Pau (IIB Sant Pau), Barcelona, Spain; 8 McKelvey School of Engineering, Washington University in St. Louis, St. Louis, MO USA; 9 Center for Neurotechnology and Neurorecovery, Massachusetts General Hospital, Boston, MA USA; 10 Department of Biostatistics and Epidemiology, University of Pennsylvania, Philadelphia, USA; 11 Department of Physics and Astronomy, University of Pennsylvania, Philadelphia, USA; 12 Institució Catalana de Recerca i Estudis Avançats (ICREA), 08015 Barcelona, Spain

**Keywords:** Cerebrovascular disease, Mean arterial pressure, Cerebral blood flow, Cerebral autoregulation, Diffuse correlation spectroscopy, Diffuse optics

## Abstract

**Background:**

The cortical microvascular cerebral blood flow response (CBF) to different changes in head-of-bed (HOB) position has been shown to be altered in acute ischemic stroke (AIS) by diffuse correlation spectroscopy (DCS) technique. However, the relationship between these relative ΔCBF changes and associated systemic blood pressure changes has not been studied, even though blood pressure is a major driver of cerebral blood flow.

**Methods:**

Transcranial DCS data from four studies measuring bilateral frontal microvascular cerebral blood flow in healthy controls (*n* = 15), patients with asymptomatic severe internal carotid artery stenosis (ICA, *n* = 27), and patients with acute ischemic stroke (AIS, *n* = 72) were aggregated. DCS-measured CBF was measured in response to a short head-of-bed (HOB) position manipulation protocol (supine/elevated/supine, 5 min at each position). In a sub-group (AIS, *n* = 26; ICA, *n* = 14; control, *n* = 15), mean arterial pressure (MAP) was measured dynamically during the protocol.

**Results:**

After elevated positioning, DCS CBF returned to baseline supine values in controls (*p* = 0.890) but not in patients with AIS (9.6% [6.0,13.3], mean 95% CI, *p* < 0.001) or ICA stenosis (8.6% [3.1,14.0], *p* = 0.003)). MAP in AIS patients did not return to baseline values (2.6 mmHg [0.5, 4.7], *p* = 0.018), but in ICA stenosis patients and controls did. Instead ipsilesional but not contralesional CBF was correlated with MAP (AIS 6.0%/mmHg [− 2.4,14.3], *p* = 0.038; ICA stenosis 11.0%/mmHg [2.4,19.5], *p* < 0.001).

**Conclusions:**

The observed associations between ipsilateral CBF and MAP suggest that short HOB position changes may elicit deficits in cerebral autoregulation in cerebrovascular disorders. Additional research is required to further characterize this phenomenon.

**Supplementary Information:**

The online version contains supplementary material available at 10.1186/s12883-021-02179-8.

## Background

Static cerebral autoregulation (CAR) maintains cerebral blood flow (CBF) during variations of the cerebral perfusion pressure [1, 2] by modulating microvascular resistance. However, static CAR may be impaired due to acute ischemic injury or in chronic disease states such as carotid steno-occlusive disease. This, in turn, may increase the vulnerability of brain tissue to ischemia and further impairments [1, 3]. In fact, the presumed impairment in static CAR provides the rationale for interventions such as volume repletion, permissive hypertension, and flat head-of-bed (HOB) positioning that are commonly performed empirically for patients with acute stroke [4, 5]. In chronic carotid steno-occlusive disease, impaired static CAR has been associated with higher risk of stroke [6, 7], cognitive decline and neurodegeneration [8–10]. There is an increasing awareness of the importance of non-invasive bedside measurements of static CAR as a biomarker to develop personalized management strategies [11]. Unfortunately, the evaluation of static CAR at the bedside is difficult.

In the past, CBF responses to the manipulation of HOB position have been employed to monitor a surrogate of static CAR impairment in patients with cerebrovascular disease [12–20]. The HOB manipulation is attractive because it is non-invasive, easy to perform, and does not require patient cooperation. Previous studies in both brain-injured and healthy subjects [12–16] showed that brief (≃minutes) HOB position changes are well tolerated and do not cause pathological alterations. On the contrary, other methods such as leg cuff occlusion/release impose additional risks in this often elderly population, for example, due to the possibility of emboli release. Changes in HOB positioning have been found to evoke macrovascular and microvascular hemodynamic responses in cerebral vasculature as measured by transcranial Doppler ultrasound (TCD) [12] and diffuse optical spectroscopy (DCS) [13–17] respectively.

The mechanisms underlying these responses are complex, reflecting a combination of factors including static CAR, venous blood return to the heart due to posture change, changes in intracranial pressure and blood volume, mean arterial pressure (MAP), and other neuroreflex mechanisms [18, 19, 21–23]. Typically, microvascular CBF correlates inversely with increases in HOB angle in patients with cerebrovascular disease, but responses are heterogeneous across subjects and between cerebral hemispheres [14–17].

Generally, the CBF changes measured from HOB angles of supine (0^°^) to 30^°^ and from 30^°^ to supine (0^°^) were not associated with neurologic deterioration, although paradoxical responses, i.e., CBF increase after HOB elevation, were identified in approximately 20% of patients with brain-injury, and obstructive sleep apnea (OSA) syndrome [14–17]. Moreover, we recently showed that the CBF response to HOB elevation in patients with acute ischemic stroke (AIS) was related to functional outcome at 3 months after the onset [20].

Curiously, CBF does not always recover to baseline after HOB position changes. In patients with OSA - a highly prevalent disorder associated with cardiovascular and cerebrovascular disease [24–27] - the failure to recover to baseline CBF after a HOB manipulation of position changes was correlated with severity and was reversed after long-term treatment [15]. To the best of our knowledge, this lack of recovery has not been evaluated in cerebrovascular disease. The assessment of CBF recovery during HOB manipulation of position changes could reflect the functional capacity of static CAR mechanisms.

The association between CBF and MAP is considered to be an indicator of CAR status. Classically, impaired CAR is characterized by CBF passively following MAP fluctuations. However, a relative “pressure-passive” relationship has been shown in healthy humans during pharmacological or orthostatic-induced changes in MAP. The relationship between CBF and systemic blood pressure change within early hours after stroke during HOB manipulation of position changes has not yet been explored [28, 29].

The assessment of CBF recovery during HOB manipulation of position changes could reflect the functional capacity of static CAR mechanisms. Acute CAR measurements could identify patients at risk for recurrent ischemic events and could also be used as a prognostic tool. This could also be applied to patients with chronic internal carotid artery (ICA) stenosis, in which static CAR evaluation could be used to identify high-risk patients who may benefit from a prophylactic carotid intervention, or could have prognostic value for predicting the future risk of stroke or cognitive decline of these patients.

Here, we investigate the recovery (supine-to-supine) of CBF after repeated HOB manipulation of position changes and test for associations in CBF changes with concurrent MAP changes. We aggregate DCS data [30–32] acquired in patients with cerebrovascular disease from four studies with similar HOB manipulation protocols. We aimed to characterize the CBF response and its association to MAP changes. We hypothesize that DCS measurements of CBF provide a biomarker of autoregulatory impairment.

## Methods

### Study population

The present work utilized data of four independent cohorts that included patients with cerebrovascular disease on whom DCS monitoring was performed using similar HOB protocols. One cohort of patients had asymptomatic severe (at least 70%) ICA stenosis or occlusion and healthy controls. The other three cohorts were comprised of patients with AIS.

Two of the AIS cohorts were studied at the Hospital of the University of Pennsylvania, USA: (1) from 2005 to 2007 [17] (PENN05–07) and (2) from 2009 to 2011 [16] (PENN09–11). Two other studies were conducted at the Stroke Unit of Hospital de la Santa Creu i Sant Pau of Barcelona: (3) a study on AIS patients from 2015 to 2017 [20] (BCN15–17), and (4) a study on ICA stenosis patients and healthy volunteers (BCN-study), both with the same HOB manipulation protocol. All protocols were approved by local internal review boards, and the participants or their legal proxies provided written consent to participate. All methods were performed in accordance with the relevant guidelines and regulations.

PENN05–07, PENN09–11 and BCN15–17 studies included patients admitted to the stroke service with AIS affecting the anterior circulation. The general exclusion criteria were intracranial hemorrhage on initial neuroimaging, and inability to lie supine for 15 min.

The BCN-study included asymptomatic patients (defined as no stroke in the territory of the stenotic artery in the preceding 6 months) with severe unilateral or bilateral extracranial ICA stenosis (at least 70%). The latter were referred to the neurosonology lab for cerebrovascular reserve testing. The exclusion criteria were bilateral inadequate temporal acoustic windows for sufficient TCD examination or the evidence of an additional intracranial stenosis in the anterior circulation. Neurologically healthy volunteers were also included in the study as controls.

The specific inclusion and exclusion criteria for each study can be found on the corresponding publications [16, 17, 20] and in Supplementary Material.

### Head-of-bed manipulation protocol

**Fig. 1:**
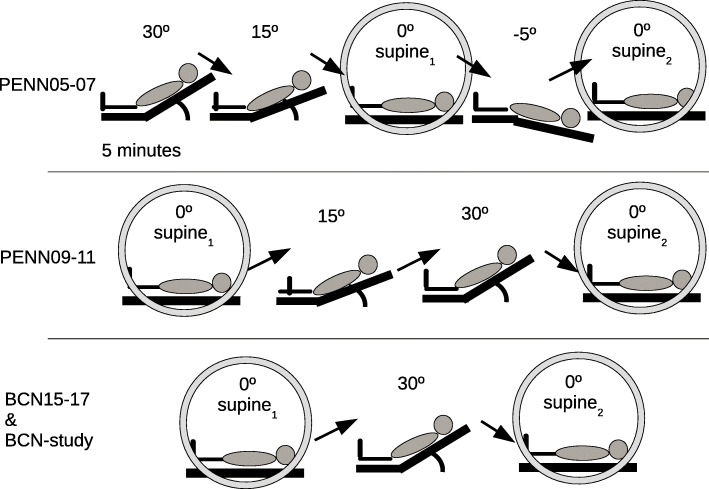
Schematic illustrating the different protocols. The supine positions used for this analysis are indicated with a circle

Different HOB position alteration protocols are illustrated in Fig. 1. All protocols involved orthostatic challenges at different HOB positions including repeated supine positions (highlighted with circles in Fig. 1). For all studies, optical data was acquired for 5 min at each position. The transition between HOB positions was noted as event markers in the data.

AIS patients were placed flat with the HOB between 0^°^ and 15^°^ according to the local clinical practice guidelines during the first 24 h after the presumed stroke onset. Afterwards, mobilization was guided by the judgment of the attending clinician.

For the PENN05–07 and PENN09–11 studies [16, 17], the study protocol was planned for three separate days and the first measurement was performed as soon as the patient was available. For the BCN15–17 study [20], the study protocol was initiated within the first 48 h after symptom onset. The protocol was repeated up-to four times at intervals of 48 h during the first week of admission as long as the patient was stable. For the BCN-study (on patients with ICA stenosis and controls), the measurements were performed once.

### Optical methods and instrumentation

The specific optical methods and instrumentation for the measurement of CBF and MAP for each study can be found in previous publications [16, 17, 20] and in Supplementary Material. Here we briefly outline the salient, common features.

For all studies, two non-invasive, optical probes were placed on the forehead bilaterally and as laterally as possible to avoid the frontal sinuses. This selection was guided by practical reasons (to avoid hair) and also since previous studies by other methods [33] and, by DCS [32], have shown that the frontal cortical area is a valid area of measurement when studying the global cerebral vasoreactivity (CVR) at the level of individual cerebral hemispheres. The probes consisted of detector fibers and a source fiber set at 2.5 cm from the detector fibers. A 2.5 cm separation provides information about the cortical cerebral hemodynamics, as previously validated [32, 34, 35].

Diffuse correlation spectroscopy has been extensively validated for measuring relative microvascular cortical CBF against other modalities [32, 35]. Recently, Giovannella et al. [36] has paved the way to calibrate for accurate absolute CBF measurements by DCS on neonates which would be applicable for adult brain measurements with appropriate means to account for the partial volume effects. Finally, we stress that, unlike near-infrared spectroscopy (NIRS), DCS is a direct measure of CBF. NIRS, on the other hand, provides surrogate measures of CBF by making assumptions about oxygen extraction and blood volume and their relationship to CBF.

The DCS system [31] employed a long coherence length laser (785 nm) single photon avalanche photo-diode detectors and a hardware auto-correlator. DCS evaluates the statistics of the diffuse laser speckles by using the auto-correlation function of the detected light intensity fluctuations. The blood flow index (BFI) of the local microvasculature is then calculated by fitting the appropriate solution of the correlation diffusion equation to the intensity autocorrelation function as previously described [30].

The changes in CBF at the second supine position were calculated by using the mean BFI at the first supine position as the baseline. The results are reported as ΔrCBF = $\overline{\left(\left(\frac{BFI_{supine2}(t)}{\overline{BFI_{supine1}}}-1\right)x100\right)}$, where $\overline{BFI_{supine1}}$ is the average of the cerebral BFI during the first supine position and BFIsupine2(t) is the continuous BFI data during the measurement at the second supine position. Up-to 1 min of the continuous BFI data was discarded for each HOB position in order to avoid bed movement artifacts.

MAP was measured continuously or at half-time of each HOB position. For the continuous MAP measurements, the first and last minutes from the analysis were discarded and the rest were used to calculate a mean of each head-of-bed (HOB) position. These data were obtained continuously by a non-invasive blood pressure monitor Finapres (Finapres Medical Systems, Arnhem, the Netherlands) device in a sub-set of patients. The mean of the MAP changes is reported as ΔMAP = $\overline{\left({MAP}_{supine2}(t)-\overline{\left({MAP}_{supine1}\right)}\right)}$. $\overline{\left({MAP}_{supine1}\right)}$ is the average of the MAP during the first supine position and MAPsupine2(t) is the continuous MAP data during the measurement on the second supine position.

When the measurements were performed at half-time of each HOB position, a manual sphygmomanometer (Omron BP785 IntelliSense Automatic Blood Pressure Monitor, Omron, Osaka, Japan) was used to measure the MAP at 2.5 min from each HOB position change. The changes are reported in the same manner as the continuous measurements.

### Clinical and imaging evaluation

The baseline examinations included the collection of demographics and vascular risk factors and a physical examination obtained by certified neurologists or senior residents under supervision who were blinded to the optical information. Diabetes mellitus, arterial hypertension and dyslipidemia were obtained for all stroke studies.

The etiologic stroke subtype was classified according to the modified Trial of Org 10,172 in Acute Stroke Treatment (TOAST) [37] criteria in two stroke studies. The extent of early ischemic changes was evaluated by the Alberta Stroke Program Early Computed Tomography Score (ASPECTS) [38] in two stroke studies. The specific clinical and imaging evaluations for each study can be found in previous publications [16, 17, 20] and in Supplementary Material.

### Statistical analysis

Quantitative clinical variables are described as a median and an interquartile range (IQR) and categorical variables as number of cases and percentages of the total (cases (percentages)). Demographic characteristics were compared across groups using either the Kruskal-Wallis test (for quantitative variables) or Fisher’s exact test (for categorical variables). If the global test was statistically significant, multiple pairwise comparisons were made using the Wilcoxon rank-sum or Fisher’s exact test to assess differences with adjustment using Holm-Bonferroni correction.

For the patient groups, each cerebral hemisphere was tagged as being “ipsilesional” or “contralesional” based on the presence of the pathology on that hemisphere. In the case of AIS patients, “ipsilesional” refers to the cerebral hemisphere where the hemisphere with cerebral ischemia was observed. In the case of ICA stenosis subjects, the categorization was by degree of the asymptomatic ICA stenosis as severe (≥70% or occlusion, “ipsilesional”) and non-severe (stenosis< 70% or absent, “contralesional”). Only patients with unilateral ICA were considered when evaluating the associations between CBF and MAP.

There are no known pathological lesions present for the healthy volunteers, we have thus randomly assigned each hemisphere measured in the controls as “side 1” or “side 2”. This was done to avoid any systematic bias by using the left/right indication.

The AIS group involved repeated measurements. In this case, linear mixed-effect models (if lack of independence in the response variable) were used for checking if the mean response of ΔrCBF or ΔMAP differed from zero, where patient identifier, the study name and the hemisphere were, if needed, the random factors for these analyses. Otherwise, simple linear models were used when the response variable was independent. Linear mixed-effect models (if there was a lack of independence in the response variable) were also used to study the association between ΔrCBF and ΔMAP, where patient identifier was the random factor. If needed in the specific model, the hemisphere and/or the study name were the covariables. Again, simple linear models were used when the response variable was independent.

For the linear models we report estimates of the mean effect along with 95% confidence intervals (95% CI). The *p*-values are reported for the hypothesis test of whether the effect is zero. Significance of specific terms was assessed using a likelihood ratio test for the full versus reduced models. A type I error of 0.05 was used to accept significance without adjustment for multiple comparisons. The “nlme” software package was used for the linear mixed-effect models implemented in R [39]. All statistical analyses were performed with R [39].

## Results

### Characteristics of the study population

Data from 114 subjects in four different studies, including 99 patients (*n* = 72 AIS and *n* = 27 ICA stenosis) and 15 healthy controls was collected. In total, 117 repeated DCS measurements during orthostatic challenges in different HOB positions were analyzed. MAP was recorded in 55 subjects, including a total of 66 measurements in 40 patients. MAP measurements were continuous in nine and at half-time in 31 patients. The control group (n = 15) had half-time MAP measurements. This information is further detailed in Table 1 and we note that the sample sizes for different variables varied because of incomplete data or artifacts.

**Table 1:** Number of subjects and total number of measurements included in each analysis

Subjects number (measurements number)	Patients				Controls	Total
	Acute ischemic stroke			ICA		
	PENN05–07	PENN09–11	BCN15–17	BCN-study		
**Total**	17 (42)	17 (21)	38 (72)	27 (27)	15 (15)	114 (177)
**Supine**_**1**_ **to supine**_**2**_ **CBF data**	15 (35)	14 (16)	38 (72)	27 (27)	15 (15)	109 (165)
**Supine**_**1**_ **to supine**_**2**_ **CBF** **ipsilesional vs contralesional** **hemisphere analysis**	15 (35)	11 (13)	36 (65)	14 (14)	–	76 (127)
**Mean arterial pressure data**	0	9 (13)	17 (39)	14 (14)	15 (15)	55 (81)
**Ipsilesional CBF data + MAP data**	0	6 (8)	14 (35)	14 (14)	–	34 (57)
**Contralesional CBF data + MAP data**	0	9 (11)	13 (31)	14 (14)	–	36 (59)

*ICA* internal carotid artery, *CBF* cerebral blood flow, *MAP* mean arterial pressure

(−) indicates that there was no specific control subject data for a particular hemisphere and specific group

The clinical characteristics of the subjects are summarized in Table 2. For the AIS patients, the severity of stroke upon arrival was not statistically significantly different across the three cohorts (NIHSS =13.5 (IQR 6, 20), *p* = 0.188). The median age of the AIS patients was 74.5 (IQR 60, 85) years but patients from AIS BCN15–17 were older than the rest (*p* = 0.006 and *p* = 0.014, for PENN05–07 and PENN09–11 studies, respectively). 53% of the subjects were female. The median time from the stroke event to the HOB manipulation measurement was 1 (IQR 0.5, 2) day but the PENN05–07 study patients were measured later from the stroke onset than the other studies (*p* = 0.044 and *p* < 0.001, for PENN09–11 and BCN15–17 studies, respectively).

**Table 2:** Demographic and clinical variables available for all groups

	Patients				Controls (*n* = 15)
	Acute ischemic stroke			ICA (*n* = 27)	
	PENN05–07 (*n* = 17)	PENN09–11 (*n* = 17)	BCN15–17 (*n* = 38)	BCN-study	
**Age;** **years median,** **(interquartile range)**	59 (53, 75)	62 (60, 64)	83 (69, 88)	68 (64, 72)	28 (28, 33)
**Females, n (%)**	10 (59%)	7 (41%)	21 (55%)	4 (15%)	6 (40%)
***Diabetes mellitus, n (%)***	8 (26.7)	–	8 (26.7)	16 (59)	1 (7)
**Hypertension, n (%)**	33 (86.8)	15 (88.2)	33 (86.8)	20 (74)	0 (0)
**Dyslipidemia, n (%)**	19 (50)	14 (82.4)	19 (50)	26 (96)	0 (0)
**NIHSS on admission**	15 (6, 20)	9 (5, 13)	18 (7, 20)	–	–
**Admission ASPECTS**	–	7 (4.3, 9.7)	9 (6, 10)	–	–
**Days from stroke onset**	2 (2, 3)	1 (1, 1)	1 (0.5, 1)	–	–
**TOAST, n (%)**					
**LAA**	3 (18)	–	5 (13)	–	–
**CE**	7 (41)	–	13 (34)	–	–
**Other**	7 (41)	–	2 (5)	–	–
**Undef.**	0 (0)	–	18 (47)	–	–

*AIS* acute ischemic stroke, *ICA* internal carotid artery, *NIHSS* National Institutes of Health Stroke Scale, *ASPECTS* Alberta Stroke Program Early Computed Tomography Score, *TOAST* Trial of ORG 10172 in Acute Stroke Treatment, *LAA* Large artery atherosclerosis, *CE* cardioembolism, *Undef.* undefined etiology

(−) indicates that the data do not exist for a specific group. Values are median and interquartile range or the number and proportion

The ICA stenosis patients were 68 (IQR 64, 62) years old and 15% of them were female. All ICA stenosis patients were asymptomatic. ICA stenosis was unilateral in 16 (59%) subjects, and bilateral in 11 (41%). A prior ischemic stroke event (more than 6 months before the measurement) was recorded in seven (35%) of the patients.

Both AIS and ICA stenosis patients were older than the healthy controls (*p* < 0.001 and *p* < 0.001, respectively). Fewer female ICA stenosis subjects were included compared to the AIS study (*p* < 0.001) and the controls (*p* = 0.002). No other demographic differences were observed.

CBF and MAP responses to HOB manipulation of position changes from the first to second supine position.

**Fig. 2:**
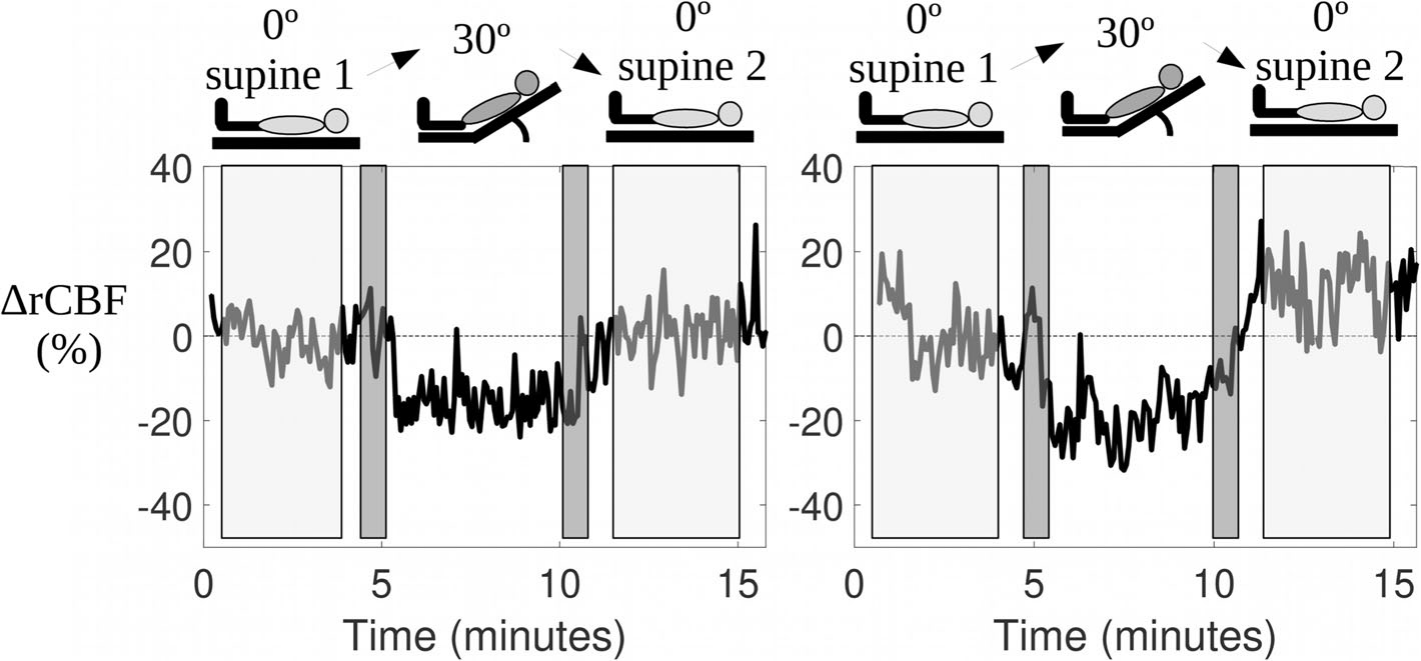
Representative microvascular CBF response to a head-of-bed position alteration. Representative microvascular CBF response (vertical axis) to a head-of-bed position alteration versus time of two different internal carotid artery stenosis patients. The periods in light gray are averaged for further analysis and dark gray shades show the transition periods. The subject on the left panel showed a CBF response that recovered back to the baseline levels whereas the other subject (right) did not

Figure 2 shows representative results for CBF changes during the head-of-bed position challenge measured by DCS from two ICA patients.

As shown in Table 1, repeated measurements from the same subjects were also assessed. The complete optical data of *n* = 109 subjects and *n* = 165 repeated measurements were included for further analysis while some data was discarded due to incomplete collection or quality degradation due to excessive patient motion.

**Fig. 3:**
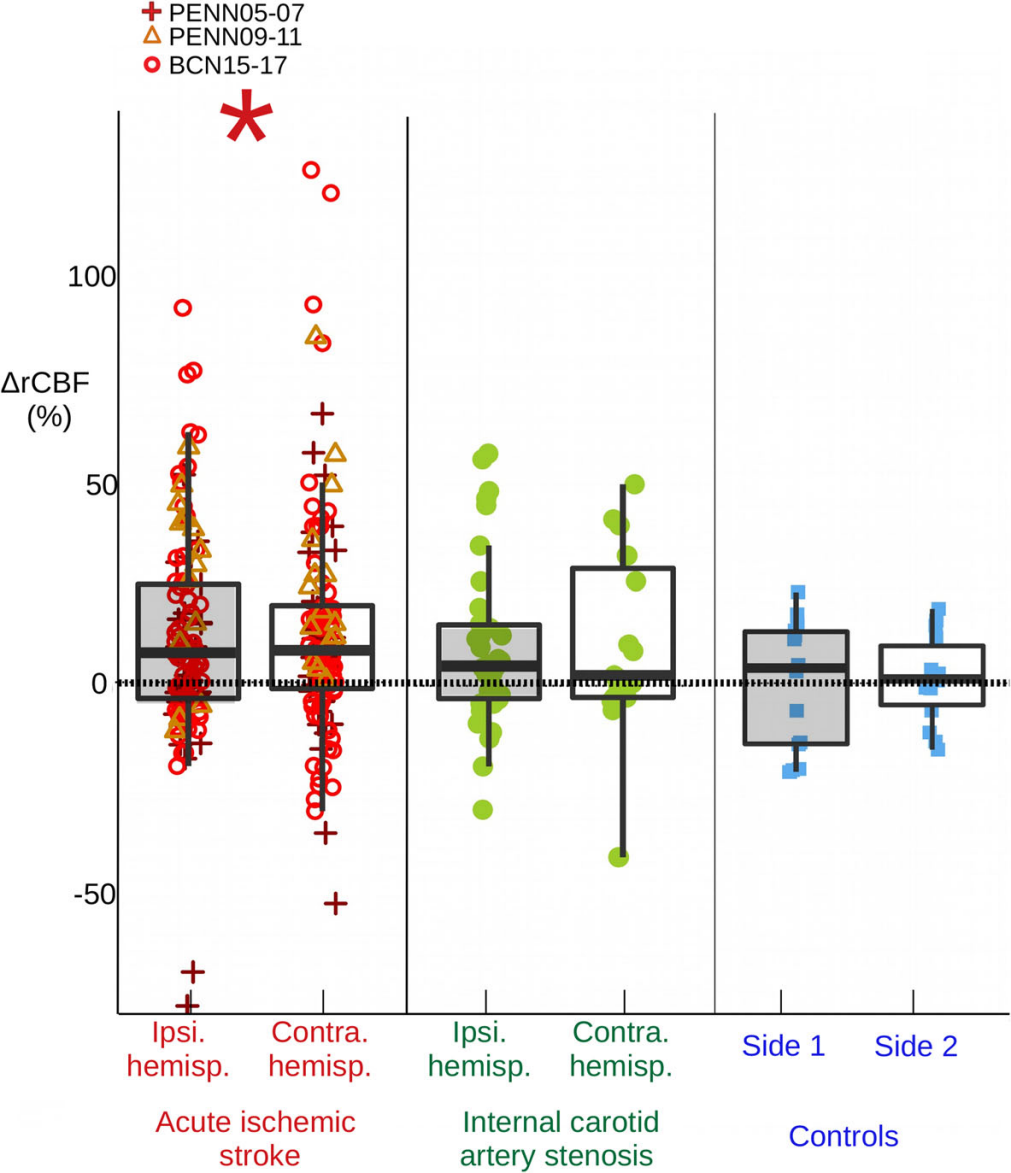
Cerebral blood flow response to the transition from the first to second supine position for all cohorts. Cerebral blood flow response (ΔrCBF) to the transition from the first to second supine position for each hemisphere for all cohorts of patients and controls is shown. 67 patients and 123 measurements were included for the acute ischemic stroke group, 27 patients for the internal carotid artery stenosis group, and 15 healthy controls. Classic boxplots and the mean ΔrCBF data point color-coded for each cohort are shown. (*) indicates that the mean response differed from zero. Ipsi. hemisp. = ipsilesional hemisphere; Contra. hemisp. = contralesional hemisphere

In AIS patients (*n* = 67), ΔrCBF was 9.6% [6.0, 13.3] [mean, 95% confidence interval] and did not recover back to its baseline values (*p* < 0.001) as shown in Fig. 3. In contrast, in controls (*n* = 15), ΔrCBF was 0.3% [− 4.7, 5.4], and it recovered back to baseline values (*p* = 0.890). We have compared the two groups using the data derived from the same protocol (BCN15–17 and the controls of the BCN-study) since the other protocols on ischemic stroke did not have controls. The ΔrCBF was different between the AIS (*n* = 38) and control (*n* = 15) groups (*p* = 0.038).

In ICA stenosis patients (*n* = 27), ΔrCBF was 8.6% [3.1, 14.0] and it did not recover back to its baseline values (*p* = 0.003) as shown in Fig. 3. Furthermore, we have compared ΔrCBF between the ICA stenosis and control subjects (*n* = 15) (BCN-study protocol) and no difference was found (*p* = 0.082).

The observed ΔrCBF responses did not differ between hemispheres, for example due to the presence of either acute stroke (*p* = 0.588) or unilateral ICA stenosis (*p* = 0.936). Only subjects with optical data available from both hemispheres were included in this sub-analysis (*n* = 62).

**Fig. 4:**
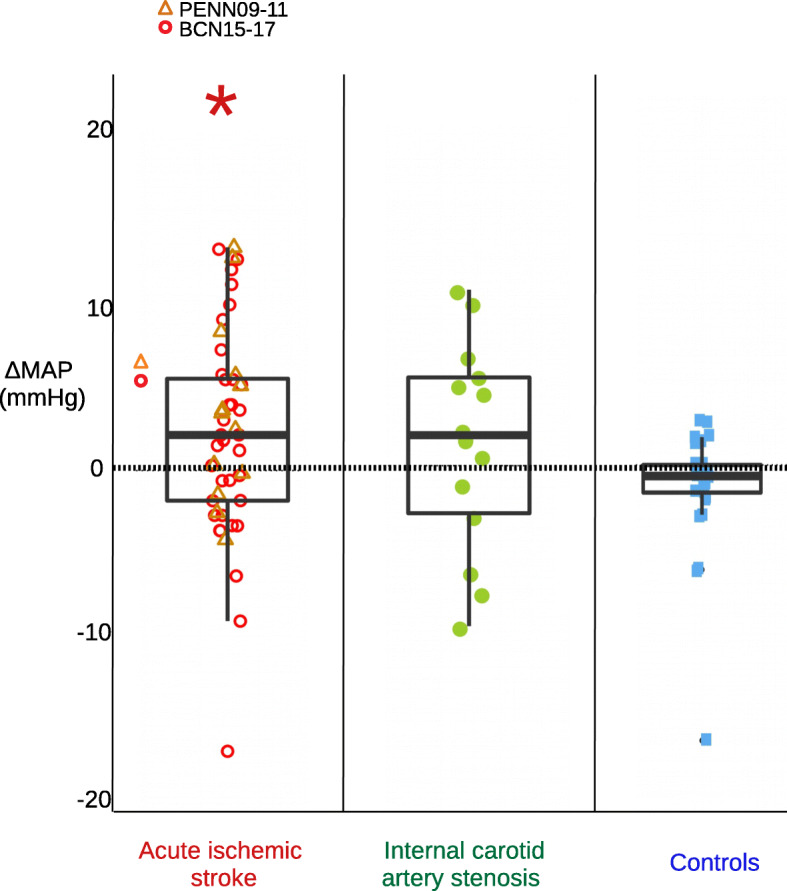
Mean arterial pressure response to the transition from the first to second supine position for all cohorts. Mean arterial pressure response (ΔMAP) to the transition from the first to second supine position for all cohorts of patients and for controls is shown. 26 (52) -number of patients (total number of measurements performed)- were included for the acute ischemic stroke group, 14 (14) for the internal carotid artery stenosis group, and 15 (15) healthy controls. Classic boxplots and the mean ΔrCBF data point color-coded for each cohort group are shown. (*) indicates that the mean response differed from zero

A significant MAP increase of 2.6 mmHg [0.5, 4.7] was observed in AIS patients (*n* = 26, *p* = 0.018), and a non-significant change of 1.4 mmHg [− 2.3, 5.1] was observed in ICA stenosis patients (*n* = 14, *p* = 0.431), and also in controls, − 1.9 mmHg [− 4.6, 0.9] (n = 15, *p* = 0.165), Fig. 4.

### Correlation between relative cerebral blood flow changes and relative mean arterial pressure changes during HOB manipulation of position changes

**Fig. 5:**
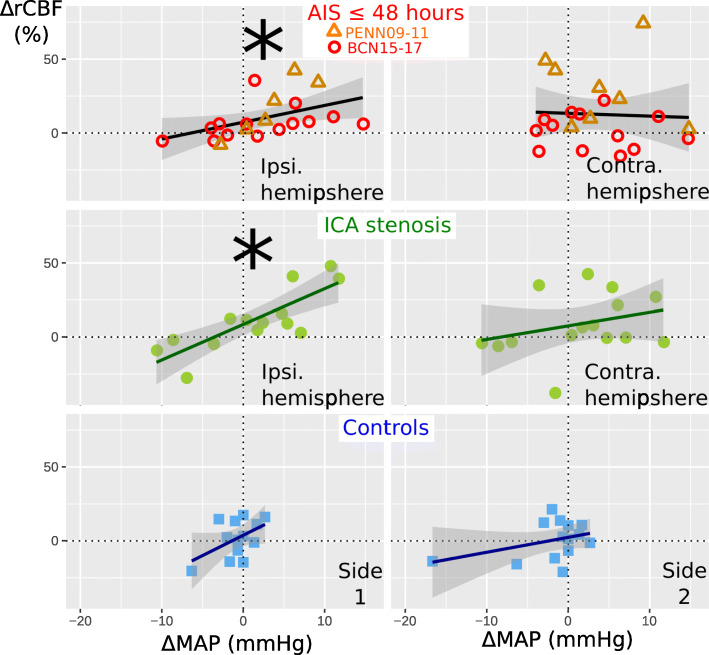
Cerebral blood flow response versus relative mean arterial pressure change. Cerebral blood flow response (ΔrCBF) for the transition from the first to the second supine position versus relative mean arterial pressure change (ΔMAP) in the ipsilesional hemisphere (left), and in the contralesional hemisphere (right) for the measurements performed on AIS in the first 48 h after stroke, ICA stenosis patients and controls. For more details about the statistical results, see the text. Linear model fit and 95% confidence intervals (in grey) are plotted. (*) indicates a statistically significant linear model. Ipsi. = ipsilesional; contra. = contralesional

In patients with AIS, ΔrCBF measured in the ipsilesional hemisphere was positively associated with ΔMAP as 6.0%/mmHg [− 2.4, 14.3] (*p* = 0.038, *n* = 20) in the early hours (≤ 48 h) from the stroke onset, Fig. 5. By contrast, the contralesional hemisphere did not exhibit a significant association being 3.1%/mmHg [− 10.0, 16.2] (*p* = 0.600) between ΔrCBF and ΔMAP parameters. However, there were no detectable differences in the MAP/CBF association between the two hemispheres of the same patients (*p* = 0.467, *n* = 22) possibly due to the high variability in the measurement and limited sample size.

In patients with ICA stenosis, the CBF changes that were observed on the ipsilesional hemisphere were associated with ΔMAP as 11.0%/mmHg [2.4, 19.5] (*p* < 0.001, *n* = 14). Again, the contralesional hemisphere did not exhibit a similar association being 8.6%/mmHg [− 5.9, 23.1] (*p* = 0.303, *n* = 14) between both parameters. This did not imply that there were intra-subject differences (*p* = 0.682). Similarly, there was no association between CBF and MAP in control subjects (*p* = 0.605).

## Discussion

We have aggregated ΔrCBF data that was acquired during HOB manipulation protocols from hundred and four patients with cerebrovascular disease to examine the variation and relationship of CBF and MAP upon returning to the supine position after orthostatic challenges.

We have identified a significant increase in CBF from the first supine position to the second supine position in AIS and ICA stenosis patient groups (*p* < 0.001 and *p* = 0.003, respectively) but not in controls (*p* = 0.890). This finding is similar to prior observations in patients with moderate and severe obstructive sleep apnea (OSA) [15]. This is an encouraging finding for further study on these populations since previous work [15] has shown that it was related to disease severity and long-term effects. If validated in these populations, it could be a prognostic biomarker.

A significant increase of ΔMAP was observed in AIS patients from the first supine position to the second supine position (*p* = 0.018) which was not observed in the ICA patients nor in the control group (*p* > 0.05). This raises the question of whether there is a significant impairment of the cerebral autoregulation which may imply a risk situation for the patient. We note that it is also known that AIS itself affects the resting arterial blood pressure if the compensatory mechanisms are damaged [2]. Detrimental conditions may occur even without any challenges [40, 41], i.e. cerebral perfusion may passively follow the variations in MAP since normal autoregulatory mechanisms are impaired [40, 41].

The physiological basis underlying the lack of return to the baseline of CBF and MAP after a brief period of HOB position changes is unclear. One possible explanation is that patients with cerebral pathology need more than 5 min in each HOB position to stabilize their cerebral and systemic hemodynamics. This view is consistent with the TCD measurements by Urbano et al [42] who explored the effect of stronger orthostatic challenges (standing to squatting position) in patients with moderate or severe OSA who also often have impaired static CAR [43]. Patients with OSA exhibited significantly slower (~seconds) recovery rates for MAP, CBF velocity, and cerebrovascular conductance compared to the control group. More research is needed to understand the temporal dynamics (from seconds to hours) following orthostatic challenges in healthy subjects and patients with compromised static CAR.

It is also interesting to compare our results with those of Aries et al [12] from the AIS population. That study employed TCD to measure CBF *velocity* changes in response to HOB position changes, and, also included concurrent MAP monitoring. Aries et al [12] found an increase in MAP from the first to last (fourth) supine position of the protocol; this MAP increase is similar to that of our work. Conversely, they did not observe an accompanying increase in CBF velocity, which is consistent with our prior findings in Favilla et al [16] (PENN09–11). This discrepancy may have arisen because TCD measures velocity in large arteries rather than the microvascular perfusion measured in the current study.

We have observed that the CBF changes in the ipsilesional hemisphere were associated with MAP changes in subjects with AIS studied within 48 h after stroke onset (*p* = 0.038), as well as with the group of subjects with ICA stenosis (*p* < 0.001). Notably, this association was not detected in the contralesional hemisphere of the AIS or ICA stenosis patients (*p* = 0.600 and *p* = 0.303, respectively), nor was there an association between CBF and MAP in control subjects (*p* = 0.605). The question that arises is whether this implies that impaired static CAR is hemispheric. Since the association between CBF and MAP are often considered to be an indication of impaired CAR [1, 2, 11, 29, 44], we suggest that our findings imply that both ICA stenosis and AIS patients have hemispheric static CAR impairments [1].

We note that in the case of AIS patients, the impairment appears to diminish with time since the association between CBF and MAP was observed only during the first 48 h after the stroke onset. Alternatively, this time-dependent result may be attributable to the increased heterogeneity of the patient population as the time after stroke increased. This is expected since the evolution of ischemic injury following AIS is heterogeneous. Ideally, the study should have been conducted at the hyper-acute time period, e.g. during the first 6 to 8 h after the stroke onset since the majority of the ischemic penumbra is viable during that period. However, this finding may still be relevant since some studies using imaging techniques have identified viable penumbra up to 48 h post stroke onset [45, 46]. In addition, cerebrovascular auroregulation has been associated with other mechanisms of secondary damage that occur later than the recruitment of ischemic penumbra, such as hemorrhagic transformation and cerebral edema, which also have an impact on the prognosis.

Our study has several other limitations. Unfortunately, data of ASPECT and TOAST classification is missing in one of the three cohorts. Final infarct volumes were calculated utilizing different imaging techniques and at different time points, which precludes a comparison among patients and cohorts. Even though EtCO_2_ changes have not been measured in the studies, we point out that an extensive study on a healthy population over a large age range has shown that EtCO_2_ does not change significantly during this challenge [13]. Our unpublished data (mean [range], supine-to-30: 0.046 [− 1.3,1.1] mmHg, *p* = 0.9; 30-to-supine: 0.26 [− 1.1,1.7], mmHg, *p* = 0.23) from an on-going study (currently at *n* = 72 subjects) on a similar population of acute ischemic stroke patients have confirmed that this is true for the patient population too. The minimal changes observed in some individuals are not expected to correlate with MAP. Moreover, the use of different protocols, from different centers, separated over time has increased the variability between studies. For example, the MAP assessment was continuous in a small subset but intermittent in all the others and different instruments were used. One study (PENN05–07) had a further limitation in utility due to lack of blood pressure measurements. We include this study as it provides critical data for the first result in our present analysis: the effect of return-to-supine on CBF. Furthermore, the studies cover a time period of 12 years with different protocols. The clinical care and management has evolved during this period including changes in patient demographics. Furthermore, technology has developed and our methods have also changed in this 12 year period. Despite these differences, importantly, similar results were found across studies which strengthens the results reported in this work. Therefore, our results are encouraging to suggest future multi-center studies with common protocols and similar clinical management at the point-of-care.

The ΔrCBF increase found in the contralesional hemisphere in our hypothesis generating study needs further exploration. One of the reasons of this result could be caused by small number of subjects compared to the amount of heterogeneity in the results. It is important to note that the result is hemispheric which rules out that the optical data is dominated by systemic changes due to partial volume effects. Future studies could address these findings by relying on the utilizing more recent advances in technology, by combination of DCS with near-infrared spectroscopy and by the so called fast measurements evaluating beat-to-beat hemodynamic changes [30, 32, 47, 48] which should improve data fidelity, and help to answer these and other questions in larger studies.

Currently, a routine evaluation of static CAR is not utilized in clinical practice for several reasons. Among these are the lack of a gold-standard methodology and the requirement to induce hemodynamic changes. The stimuli utilized by many methods (e.g., rapid release of thigh tourniquets or infusion of vasoactive drugs) have issues with safety and patient compliance. HOB manipulations are simpler and safer compared to these methods, even in patients with acute brain injury. In this contribution, we have shown that the increase in correlation between DCS-measured CBF and MAP during serial HOB position changes is a promising protocol to assess static CAR performance in patients with both acute and chronic cerebrovascular disease. Further studies are needed to ascertain whether this information can be used to individualize management of patients with cardiovascular disease.

## Conclusions

A brief and mild HOB challenge elicits evidence of cerebrovascular autoregulatory deficits. DCS can be used to monitor these changes at the bed-side, and after further validation, may ultimately help to individualize clinical management in AIS.

## Supplementary Information

**Additional file 1.**

## Data Availability

The datasets used and/or analyzed during the current study available from the corresponding author on reasonable request.
