# Supplementary material for: Blood flow response to orthostatic challenge identifies signatures of the failure of static cerebral autoregulation in patients with cerebrovascular disease
Source: BMC Neurol. 2021 Apr 9;21:154. doi: 10.1186/s12883-021-02179-8 (PMC8033703; doi:10.1186/s12883-021-02179-8)
Supplement: Supplementary file 1 — Additional file 1. [file 12883_2021_2179_MOESM1_ESM.pdf]

Blood flow response to orthostatic challenge identifies signatures of the failure of static cerebral autoregulation in patients with cerebrovascular disease

Clara Gregori-Pla[1], Rickson C. Mesquita[2], Christopher G. Favilla[3], David R. Busch[4], Igor Blanco[1], Peyman Zirak[1], Lisa Kobayashi Frisk[1], Stella Avtzi[1], Federica Maruccia[1,5], Giacomo Giacalone[1,6], Gianluca Cotta[1], Pol Camps-Renom[7], Michael T. Mullen[3], Joan Martí-Fàbregas[7], Luís Prats-Sánchez[7], Alejandro Martínez-Domeño[7], Scott E. Kasner[3], Joel H. Greenberg[3], Chao Zhou[8], Brian L. Edlow[9], Mary E. Putt[10], John A. Detre[3], Arjun G. Yodh[11], Turgut Durduran[1,12], and Raquel Delgado-Mederos[7]

[1]ICFO-Institut de Ciències Fotòniques, The Barcelona Institute of Science and Technology, Castelldefels (Barcelona), Spain.

[2]Institute of Physics, University of Campinas, Campinas, Brazil.

[3]Department of Neurology, University of Pennsylvania, Philadelphia, USA.

[4]Departments of Anesthesiology and Pain Management and Neurology, University of Texas Southwestern Medical Center, Dallas, USA.

[5]Neurotraumatology and Neurosurgery Research Unit (UNINN), Vall d'Hebron University Research Institute (VHIR), Universitat Autònoma de

Barcelona, Barcelona, Spain.
[6]San Raffaele Scientific Institute, Milan, Italy.
[7]Biomedical Research Institute Sant Pau (IIB Sant Pau). Department of Neurology (Stroke Unit). Hospital de la Santa Creu i Sant Pau, Barcelona, Spain.
[8]McKelvey School of Engineering, Washington University in St. Louis, St. Louis MO, USA
[9]Center for Neurotechnology and Neurorecovery, Massachusetts General Hospital, Boston MA, USA
[10]Department of Biostatistics and Epidemiology, University of Pennsylvania, Philadelphia, USA
[11]Department of Physics and Astronomy, University of Pennsylvania, Philadelphia, USA
[12]Institució Catalana de Recerca i Estudis Avançats (ICREA), Barcelona, Spain.

Supplementary material

Inclusion and exclusion criteria for each study

- PENN06-07[1] & PENN09-11[2]:

  Inclusion: patients admitted to the stroke service with radiographic evidence or clinical suspicion of acute ischemic stroke involving the frontal lobe cortex.

Exclusion: intracranial hemorrhage on initial computed tomography (CT) or magnetic resonance imaging (MRI) scan and inability to lie supine for 15 minutes.

- BCN15-17[3]:

  Inclusion: being older than 18 years, a National Institutes of Health Stroke Scale (NIHSS) score[4] of more than three at admission, radiographic evidence or clinical suspicion of acute ischemic stroke involving the frontal lobe cortex, pre-stroke modified Rankin Scale (mRS) score[5] of less than three, and written informed consent.

  Exclusion: resting heart rate less than 40 or greater than 110 beats per minute, peripheral arterial oxygen saturation less than 92% with supplementation, diagnosis of transient ischemic attack or minor stroke and acute ischemic stroke in the posterior territory.

- ICA-study:

  Inclusion of internal carotid artery (ICA) stenosis: unilateral or bilateral severe (at least 70%) carotid stenosis or occlusion of the ICA.[6]

  Exclusion of ICA stenosis: bilateral inadequate temporal acoustic windows for sufficient transcranial Doppler ultrasound examination or the evidence of an additional intracranial stenosis of the carotid siphon, of the middle cerebral artery, or of the anterior cerebral artery.

  Controls/healthy volunteers consisted of hospital employees and medical students.

  Inclusion of controls: being older than 18 years, informed consent.

  Exclusion of controls: past history of stroke, presence of carotid or middle cerebral artery stenosis or occlusion.

Optical methods and instrumentation for each study

- PENN06-07[1]:

A portable custom-built instrument was employed as described in Refs.[7,8] Near-infrared diffuse optical spectroscopy (NIRS-DOS) was also implemented, although these data are not used in the present analysis. The data acquisition was interleaved between NIRS-DOS and diffuse correlation spectroscopy (DCS). For each probe, NIRS-DOS data was acquired for half a second on one hemisphere, then DCS data was acquired for three seconds; the process was repeated on the contralateral hemisphere, giving a total measurement time per data point of seven seconds. The first minute in each position was discarded from the analysis of relative cerebral blood flow (ΔrCBF) changes due to possible movement artifacts and for avoiding systemic instabilities for this dataset. No mean arterial pressure (MAP) measurements were acquired for this study.

- PENN09-11[2]:

  Data were collected from both hemispheres every three seconds using the same instrument as in the previous study.[1] Again, the first minute in each position was discarded from the analysis of ΔrCBF changes. For the continuous MAP measurements, the first and last minutes from the analysis were discarded and the rest were used to calculate a mean of each head-of-bed (HOB) position. For this study, continuous non-invasive blood pressure monitor Finapres (Finapres Medical Systems, Arnhem, the Netherlands) device was used to measure the arterial blood pressure in a sub-set of patients.

- BCN15-17[3] & ICA-study:

  The custom built diffuse correlation spectroscopy system was previously described in Refs.[9,10] Data were collected from both hemispheres every 2.5 seconds. The first and last minutes from the analysis of ΔrCBF changes were discarded. In this study, a manual sphygmomanometer (Omron BP785 IntelliSense Automatic Blood Pressure Monitor, Omron, Osaka, Japan) at 2.5 minutes from each HOB change was used to measure the arterial blood pressure.

Clinical and imaging evaluations

- PENN06-07[1]

  Baseline examinations included the collection of demographics and vascular risk factors from verbal history and available medical records. The stroke severity was assessed with the NIHSS at admission. Stroke etiology was classified according to the *Trial of ORG 10172 in Acute Stroke Treatment* (TOAST) criteria.[11] A neurologist reviewed all available head CT and brain MRI data to determine stroke (or infarct) laterality, vascular territory, and region(s) of the brain involved. The primary indicators for radiographic evidence of stroke were hypodensity on CT and/or diffusion restriction on diffusion MRI sequences. CT and magnetic resonance angiographic data were also used to support the assessment of vascular territory involvement when being available. Vessel involvement was characterized as middle cerebral artery or anterior cerebral artery. Brain region involvement was characterized as frontal, temporal, parietal or occipital.

- PENN09-11[2]

  Baseline examinations included the collection of demographics and vascular risk factors. The stroke severity was assessed with the NIHSS at admission, at the moment of the measurement and at discharge. Stroke cause was recorded. The extent of early ischemic changes was evaluated on noncontrast CT by the Alberta Stroke Program Early Computed Tomography Score (ASPECTS).[12] Functional outcome was evaluated by the mRS.

- BCN15-17[3]

  Baseline examinations included the collection of demographics and vascular risk factors and a physical examination. The stroke severity was assessed with the NIHSS[4] at admission, at the moment of the measurement, at 24 and at 48 hours from stroke onset, and at discharge. The etiologic stroke subtype was classified according to the

modified TOAST criteria.[13] At admission, a cranial CT was performed on all patients and also a vascular imaging with either CT-angiography or color-coded duplex sonography. The extent of early ischemic changes was evaluated on noncontrast CT by the ASPECTS. Recanalization was assessed by transcranial duplex before the HOB protocol. Functional outcome was evaluated at three months by the mRS.

- ICA-study

  ICA stenosis patients: The ICA stenosis was defined as asymptomatic if no hemispheric nor retinal ischemic symptoms ipsilateral to the stenosed side had occurred in the preceding six months. Patients were referred to our neurosonology laboratory for vasoreactivity testing. The carotid stenosis or occlusion of the ICA as determined by published criteria using a carotid artery ultrasound (multifrequency linear array transducer; Aplio-XG, Toshiba, Tochigi, Japan).[6] In these patients, the grade of ICA stenosis or occlusion was further confirmed by noninvasive magnetic resonance or computed tomography angiography and, in selected cases, by digital subtraction angiography according to North American Symptomatic Carotid Endarterectomy Trial (NASCET) criteria.[14]

  Controls: The diagnostic evaluation included a review of past mediacl history and carotid and transcranial duplex ultrasound exam to rule out carotid or intracranial steno-occlusive lesions.

References

[1] Durduran T, Zhou C, Edlow BL, Yu G, Choe R, Meeri N, et al. Transcranial optical monitoring of cerebrovascular hemodynamics in acute stroke patients. Opt Express. 2009;17(5):3884–3902.

[2] Favilla CG, Mesquita RC, Mullen M, Durduran T, Lu X, Kim MN, et al. Optical bedside monitoring of cerebral blood flow in acute ischemic stroke patients during head-of-bed manipulation. Stroke. 2014 mar;45((5)):1269–1274.

[3] Gregori-Pla C, Blanco I, Camps-Renom P, Zirak P, Serra I, Cotta G, et al. Early microvascular cerebral blood flow response to head-of-bed elevation is related to outcome in acute ischemic stroke. Journal of Neurology. 2018;.

[4] Brott T, Adams HPJ, Olinger CP, Marler JR, Barsan WG, Biller J, et al. Measurements of acute cerebral infarction: a clinical examination scale. Stroke. 1989;20(7):864–870.

[5] Van Swieten JC, Koudstaal PJ, Visser MC, Schouten H, Van Gijn J. Interobserver agreement for the assessment of handicap in stroke patients. Stroke. 1988;19(5):604–607.

[6] Grant EG, Benson CB, Moneta GL, Alexandrov AV, Baker JD, Bluth EI, et al. Carotid artery stenosis: grayscale and Doppler ultrasound diagnosis–Society of Radiologists in Ultrasound consensus conference. Ultrasound Q. 2003;19(4):190–198.

[7] Cheung C, Culver JP, Takahashi K, Greenberg JH, Yodh AG. In vivo cerebrovascular measurement combining diffuse near-infrared absorption and correlation spectroscopies. Phys Med Biol. 2001;46(8):2053–2065.

[8] Durduran T. Non-invasive measurements of tissue hemodynamics with hybrid diffuse optical methods; 2004.

[9] Delgado-Mederos R, Gregori-Pla C, Zirak P, Blanco I, Dinia L, Marín R, et al. Transcranial diffuse optical assessment of the microvascular reperfusion after thrombolysis for acute ischemic stroke. Biomed Opt Express. 2018;9(3):1262.

[10] Zirak P, Delgado-Mederos R, Martí-Fàbregas J, Durduran T. Effects of acetazolamide on the micro- and macro-vascular cerebral hemodynamics: a diffuse optical and transcranial doppler ultrasound study. Biomed Opt Express. 2010;1(5):1443–1459.

[11] Adams HP, Bendixen BH, Bendixen BH, Kappelle LJ, Kappelle LJ, Biller J, et al. Classification of subtype of acute ischemic stroke. Stroke. 1993;23(1):35–41.

[12] Barber PA, Demchuk AM, Zhang J, Buchan AM. Validity and reliability of a quantitative computed tomography score in predicting outcome of hyperacute stroke before thrombolytic therapy. Library (Lond). 2000;.

[13] Han SW, Kim SH, Lee JY, Chu CK, Yang JH, Shin HY, et al. A new subtype classification of ischemic stroke based on treatment and etiologic mechanism. Eur Neurol. 2007;57(2):96–102.

[14] of North American Symptomatic Carotid Endarterectomy Trial C. Beneficial effect of carotid endarterectomy in symptomatic patients with high-grade carotid stenosis. N Engl J Med. 1991;325(7):445–53.
